# Improving antioxidant and antiproliferative activities of colla corii asini hydrolysates using ginkgo biloba extracts

**DOI:** 10.1002/fsn3.587

**Published:** 2018-03-09

**Authors:** Xiaobing Xu, Shangwei Guo, Xianghui Hao, Hui Ma, Yanping Bai, Yaqin Huang

**Affiliations:** ^1^ State Key Laboratory of Chemical Resource Engineering Beijing Laboratory of Biomedical Materials Beijing University of Chemical Technology Beijing China; ^2^ Dong E E Jiao Co., Ltd Dong'e China; ^3^ China‐Japan Friendship Hospital Beijing China

**Keywords:** antioxidant, antiproliferative activity, colla corii asini hydrolysates, ginkgo biloba extracts

## Abstract

Colla corii asini hydrolysates (ACCH) and ginkgo biloba extracts (EGb) possess more potent antioxidant effects when used in combination than when used alone. The mixture of ACCH and EGb at a dose ratio of 20:4(w:w) showed the highest radical scavenging activity with IC_50_ of 0.17 ± 0.01, 0.43 ± 0.02 and 1.52 ± 0.07 mg/ml against DPPH, ABTS and HO ^·^ free radicals, respectively. Furthermore, the inhibition of breast cancer cells MCF‐7 and MDA‐MB‐231 proliferation increased when these cell lines were treated with a combination of ACCH and EGb for 72 hr, with IC_50_ of 4.32 ± 0.12 mg/ml and 0.39 ± 0.01 mg/ml, respectively. The findings indicated that the mixtures of ACCH and EGb could be used to prevent and treat some diseases caused by the excessive free radicals, especially cancer. Therefore, the mixtures of ACCH and EGb might serve as a natural source of desirable antioxidant and anticancer agents for the nutraceutical and pharmaceutical industries.

## 1 INTRODUCTION

Cancer is one of the leading causes of death in humans (Li et al., 2017). Many studies have showed that the excessive free radicals produced under oxidative stress lead to oxidative damage to key functional elements including cellular lipids, DNA, and proteins, which is associated with the pathogenesis of inflammation‐related cancers (Chi, Hu, Wang, Li, & Ding, 2015; Ko et al., 2016). Also, Valko, Izakovic, Mazur, Rhodes, and Telser (2004) and Asaduzzaman, Tania, Zhang, and Chen (2010) have found that there is a certain link between antioxidant and anticancer. Therefore, antioxidants have been proposed as candidates for the prevention and treatment of cancers (Pan, Zhao, Hu, Chi, & Wang, 2016; Sila & Bougatef, 2016). However, the use of certain synthetic antioxidants and anticancer drugs has been of concern due to their toxicity and side effect (Kwak, Seo, & Lee, 2009). Thus, discovery of natural antioxidants with the property to specifically target cancer cells without toxicity to normal cells as alternative to synthetic anticancer drugs can be of great significance.

Much attention has been placed on protein hydrolysates because of their low cost, high nutritional value, excellent biocompatibility, and safe without toxic side effect (Farvin et al., 2016; Karnjanapratum, O'Callaghan, Benjakul, & O'Brien, 2015; Sah, Vasiljevic, Mckechnie, & Donkor, 2014). Colla corii asini hydrolysate (ACCH) is a protein hydrolysate and is obtained by hydrolyzing colla corii asini. Colla corii asini is a solid glue prepared by stewing and concentrating from *Equus asinus* L. donkey hide (Wu et al., 2007). Many pharmacological studies have confirmed that colla corii asini has certain therapeutic effects, including hemostasis, anti‐aging, antitumor, immunity‐enhancing, antifatigue, and so on (Li et al., 2013; Tian et al., 2017; Wang et al., 2012; Wang, Ru, et al., 2014). The protein content in colla corii asini is about 80% by weight. Low molecular weight ACCH is expected to be more efficacious than colla corii asini itself (Wu et al., 2016). To maximally utilize ACCH, the antioxidant of ACCH need to be further improved.

Certain plant extracts, especially the phenolics and flavonoids, have been identified as excellent antioxidants with strong activity in scavenging free radicals (Lin et al., 2017; Wang, Zhao, et al., 2014). Ginkgo biloba extracts (EGb), one of the oldest herbal medicines, have biological effects such as scavenging free radicals, lowering oxidative stress, reducing neural damages, reducing platelet aggregation, anti‐inflammation, antitumor activities, and anti‐aging (He et al., 2014). EGb has been used as antioxidant additive to certain food products due to its strong antioxidant activity. For example, EGb was added to gelatin‐based films and greatly improved the antioxidant property of gelatin‐film (Li, Miao, Wu, Chen, & Zhang, 2014). These works inspired us to explore the interactions between ACCH and EGb, and further improve the antioxidant of ACCH.

Our study has confirmed the synergistic effect between ACCH and EGb using isobolographic analysis. Moreover, this study has shown that ACCH mixed with EGb has strong cytotoxicity toward MCF‐7 and MDA‐MB‐231 cell lines in a dose‐dependent manner. Therefore, we surmise the mixture of ACCH and EGb may be potentially useful to prevent and treat some diseases linked with oxidative stress, e.g., cancer.

## 2 MATERIALS AND METHODS

### 2.1 Materials

Colla corii asini was obtained from Shandong Dong‐E‐E‐Jiao Co., Ltd. (Dong‐e, China). Ginkgo biloba extracts (EGb) were purchased from Chinese Academy of Food and Drug Control. 1,1‐Diphenyl‐2‐picryl‐hydrazyl (DPPH) and 2,2‐azino‐bis (3‐ethyl benzothiazoline‐6‐sulfonic acid) diammonium salt (ABTS) were purchased from Ye source Biotechnology Co., Ltd. (Shanghai, China). L929 was obtained from Shanghai Health Bo Biological Medicine Technology Co., Ltd. (Shanghai, China). MCF‐7, MDA‐MB‐231, were obtained from Beijing Jia Mei Technology Co., Ltd. (Beijing, China). Cell Counting Kit‐8 (CCK‐8) was purchased from DOJIODO Laboratories (Japan). Other chemicals and reagents were of analytical grade commercially available.

### 2.2 Preparation of colla corii asini hydrolysates (ACCH)

Colla corii asini used in this study was supplied by Shandong Dong‐E‐E‐Jiao Co., Ltd. The colla corii asini was hydrolyzed by Alcalase, which was carried out at a temperature of 50°C, pH 8.0, E:S of 0.4 (w/w) and for 3 hr. The hydrolysis was terminated by boiling at 100°C for approximately 15 min. After being filtered and dried, the obtained colla corii asini hydrolysate powder was used in subsequent investigations.

### 2.3 Amino acid composition analysis

The amino acids contents in colla corii asini hydrolysates (ACCH) were measured using Hitachi amino acid analyzer L‐8900.

### 2.4 Determination of DPPH scavenging activity

A slightly modified DPPH method (Brand‐Williams, Cuvelier, & Berset, 1995) was used for radicals scavenging evaluation. DPPH radical solution was prepared by dissolving 7.89 mg of DPPH in 100 ml of ethanol. Briefly, instead of reading samples spectrophotometrically directly at 517 nm, the assay was performed in a 96‐well flat‐bottom microplate with 100 μl of DPPH solution and 100 μl of the tested sample. The plate was then covered and left in the dark for 60 min at room temperature. The absorbance was read at 517 nm with a microplate reader (SpectraMax M2^e^ Molecular Devices). All samples were tested in triplicate. DPPH free radicals scavenging activity of each solution was calculated using the following equation:

(1) Inhibition rate(%)=[1−(Ai−Aj)/A0]∗100%,

where A0 was the absorbance of distilled water (100 μl) and DPPH radical solution (100 μl), Ai was the absorbance of the tested sample (100 μl) and DPPH radical solution (100 μl), and Aj was the absorbance of the tested sample (100 μl) and ethanol (100 μl).

The absorbance of a tested sample is inversely related to its DPPH radical scavenging activity. The IC_50_ value of an antioxidant was the effective concentration of the antioxidant to scavenge 50% of the DPPH and calculated from the radical scavenging activity graph.

### 2.5 Determination of ABTS scavenging activity

The ABTS free radical scavenging capacity assay was carried out using the modified method reported earlier (Arnao, Cano, & Acosta, 2001). The ABTS^·+^ solution was prepared by mixing potassium persulfate (2.45 mmol/L) with ABTS solution (7 mmol/L). Before the test, the mixtures were kept for 12–16 hr at room temperature in a dark environment. Then, the ABTS^·+^ solution was diluted with a pH 7.4 phosphate buffered saline solution to an absorbance of 0.70 ± 0.02 at 734 nm. Briefly, 10 μl of a test sample was added to 190 μl of the ABTS^·+^ radical solution in a 96‐well flat‐bottom microplate. The plate was then covered and left in the dark for 15 min at room temperature. The absorbance was read at 734 nm with a microplate reader (SpectraMax M2^e^ Molecular Devices). All samples were tested in triplicate. The calculation method is similar to DPPH free radical scavenging assay. The results were expressed by IC_50_ values (the effective concentrations at which 50% of the ABTS radicals were scavenged).

### 2.6 Determination of HO^·^ scavenging activity

The hydroxyl radical scavenging activity of ACCH, EGb and their mixtures were measured according to the method of Chi et al. (2015). First, 1.0 ml of a 1.865 mmol/L 1,10‐phenanthroline solution and 2.0 ml of the sample were mixed in a screw‐capped tube. Then, 1.0 ml of a FeSO_4_·7H_2_O solution (1.865 mmol/L) was added to the mixture. The reaction was initiated by adding 1.0 ml of H_2_O_2_ (0.03%, v/v). The tube was left in a water bath for 60 min at 37°C. The absorbance was read at 536 nm with a spectrophotometer (TU‐1810). All samples were tested in triplicate. The hydroxyl radical scavenging activity was calculated using the following formula:

(2) Inhibition rate(%)=[(As−An)/(Ab−An)]∗100%,

where As was the absorbance of the sample, An is the absorbance of the negative control (distilled water instead of the sample), and Ab was the absorbance of the blank (without H_2_O_2_).

### 2.7 Determination of antiproliferative activity

The antiproliferative activity of the samples was evaluated in vitro using an CCK‐8 assay (Ma et al., 2013) and was expressed as the half‐inhibitory concentration (IC_50_), defined as the test sample concentration that is half of the concentration having the maximal antiproliferative activity. Briefly, cancer cells were seeded at a density of 1 × 10^4^ per well in 96‐well plate for 24 hr at C in a 5% CO_2_ incubator. Then, the cells were treated with different samples at different concentrations for 72 hr. Untreated cells were used as a negative control. Then, 10 μL per well of CCK‐8 was added and incubated with the plates for 1 hr. The absorbance of each cell was read at a wavelength of 450 nm with a microplate reader. The percent of cell viability was calculated as follows:

(3) Cell viability(%)=[(As−Ab)/(An−Ab)]∗100%,

where Ab was the absorbance of cck‐8 solution(10 μl) and medium (90 μl), An was the absorbance of cck‐8 solution (10 μl) and medium with cells (90 μl), and As was the absorbance of cck‐8 solution (10 μl), medium with cells and the tested sample (90 μl).

### 2.8 Isobolographic analysis

We used isobolographic analysis (Wang, Wang, & Liu, 2015) to investigate the three types of interactions (synergistic, additive or antagonistic/negative) that may occur for compounds of ACCH and EGb at the selected dose ratios (20:1, 20:2, 20:4 w/w). The interaction index (λ) between ACCH and EGb was calculated using the following equation:

(4) (a/A)+(b/B)=λ,

where A and B are the doses of substance A (alone) and B (alone), respectively, that give a specified effect; a and b are the combination doses that produce the same effect. If λ = 1, the interaction is additive; if λ < 1, the interaction is synergistic; and if λ > 1, the interaction is antagonistic.

### 2.9 FT‐IR analysis

The interaction of ACCH with EGb was observed using an FTIR‐ATR spectrometer. The scans were carried out in a spectral range varying from 500 cm^−1^ to 4,000 cm^−1^.

### 2.10 Statistical analysis

All data were collected in triplicate (*n* = 3) and reported as mean ± standard deviation (*SD*). ANOVA and the least significant difference tests were performed using a spss computer program (SPSS 22.0, IBM, USA) to identify the difference between values. Statistical significance was established at *p* < .05.

## 3 RESULTS AND DISCUSSION

### 3.1 Amino acid composition analysis

Table 1 shows amino acid composition of ACCH. ACCH was rich in Glu, Gly, Arg, Pro and Hypro, which accounted for 138.35, 130.50, 114.45, 117.26 and 125.45 residues/1,000 residues, respectively. In addition, ACCH contained high contents of hydrophobic amino acids, such as Ala (7.13%), Val (2.48%), Lys (4.98%), Pro (11.73%), Leu (3.59%), Ile (1.64%), Met (0.89%) and Gly (13.05%).

**Table 1:** Amino acid composition of colla corii asini hydrolysate (ACCH; expressed as residues/1,000 residues)

Amino acids	ACCH
Asp	64.86
Thr	18.85
Ser	34.00
Glu	138.35
Gly	130.50
Ala	71.25
Val	24.80
Met	8.86
Ile	16.38
Leu	35.91
Tyr	8.64
Phe	31.42
Lys	49.82
His	9.20
Arg	114.45
Pro	117.26
Hypro	125.45

Amino acid composition has been reported to influence the antioxidant activity of protein hydrolysates (Zhou et al., 2012; Zou, He, Li, Tang, & Xia, 2016). Hydrophobic amino acids (Ala, Val, Ile, Leu, Tyr, Phe, Pro, Met, Lys) confer antioxidant activity to protein hydrolysates owing to the abundance of their electrons that can be donated to quench free radicals (He, Girgih, Malomo, Ju, & Aluko, 2013). Moreover, hydrophobic amino acids can enhance the solubility of protein hydrolysates in lipids, thus increasing their interaction with free radicals (Najafian & Babji, 2014). Aromatic amino acids (Tyr, Phe) have been credited to enhance the potency of radical scavenging activity by donating protons to stabilize electron‐deficient radicals while retaining their own stability through resonance structures (Kimatu et al., 2017). Above antioxidant‐related amino acids are rich in ACCH, suggesting that ACCH may have potential antioxidant ability.

### 3.2 Antioxidant activity of ACCH and EGb

The antioxidant activity of drugs can be attributed to different mechanisms, including DPPH^·^ scavenging activity, ABTS^·+^ scavenging activity and HO^·^ scavenging activity, or a combination of these properties (Hu et al., 2017). As shown in Table 2, the antioxidant capacity of ACCH and EGb were measured using three antioxidant assays (DPPH^·^, ABTS^·+^ and HO^·^ scavenging activity assays). For DPPH^·^, ABTS^·+^ and HO^·^ scavenging activity assays, the antioxidant activity was expressed by IC_50_. The DPPH radical scavenging activity is a substrate way to evaluate antioxidants because DPPH can be scavenged when it encounters a proton‐donating substance (Zhuang, Tang, & Yuan, 2013). As shown in Table 2, both ACCH and EGb have ability to quench DPPH radicals. However, EGb (DPPH_IC50_ = 0.04 ± 0.01 mg/ml) has stronger DPPH radicals scavenging activity than ACCH (DPPH_IC50_ = 8.87 ± 0.30 mg/ml).

**Table 2:** Antioxidant activity of colla corii asini hydrolysate (ACCH) and ginkgo biloba extracts (EGb)

Samples	IC_50_ values (mg/ml)		
	DPPH^·^	ABTS^·+^	HO^·^
ACCH	8.87 ± 0.30^a^	4.53 ± 0.15^a^	4.40 ± 0.12^a^
EGb	0.04 ± 0.01^b^	0.11 ± 0.01^b^	0.51 ± 0.02^b^

Means in the same column with the same letters are not significantly different (*p* > .05). Data are expressed as means ± standard errors (*n* = 3).

ABTS^·+^ scavenging activity is one of the most widely methods used to measure antioxidant activity, which is related to hydrogen donating or chain breaking properties (Correa et al., 2011). Table 2 shows that EGb (ABTS_IC50 _= 0.11 ± 0.01 mg/ml) has stronger ABTS radicals scavenging activity than ACCH (ABTS_IC50 _= 8.87 ± 0.30 mg/ml), which corresponded with the DPPH measurement.

It has been confirmed that HO^·^ is a highly damaging species in free radical pathology and is capable of attacking and destroying almost all biomolecules (amino acids, proteins, lipids, and DNA) in living cells (Chi et al., 2015; Je, Byun, & Kim, 2007). Therefore, it is very important to remove excess HO^·^ in human for defensing against various diseases. Measuring HO^·^ scavenging ability could provide useful information about the antioxidant activities of the samples. As shown in Table 2, ACCH and EGb exhibited high scavenging activities against HO^·^ with dose–effect relationships and IC_50_ of 4.40 ± 0.12 and 0.51 ± 0.02 mg/ml, respectively.

ACCH contains hydrophobic amino acids (Ala, Leu, Lys), which confer antioxidant activity to ACCH owing to the abundance of their electrons that can be donated to quench free radicals. Met in ACCH has the ability to donate its sulfur hydrogen and therefore is considered as an efficient radical scavenger (Kimatu et al., 2017). In addition, EGb showed remarkable antioxidant activity because EGb can terminate the radical chain reaction by donating electrons or protons to the radicals (Li et al., 2014).

### 3.3 Antioxidant activity of the mixture of ACCH and EGb and the interaction analysis

**Figure 1:**
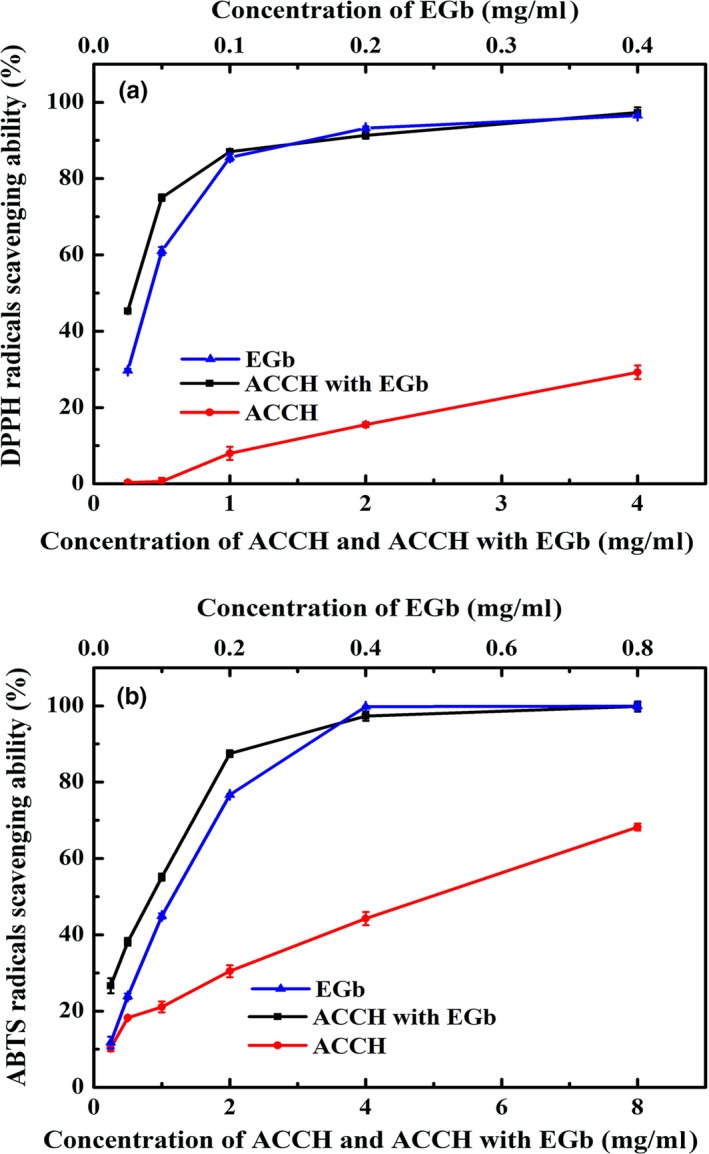
Antioxidant activities of colla corii asini hydrolysates (ACCH), ACCH with EGb and EGb as determined by DPPH radical scavenging (A), ABTS free radical scavenging (B). All data are presented as the mean ± *SD* of triplicate results

The DPPH and ABTS radical scavenging activities of ACCH, EGb, and ACCH combined with EGb are shown in Figure 1A,B, respectively. The DPPH radical scavenging activity increased with increasing concentration of ACCH, EGb, and ACCH combined with EGb (ACCH: EGb = 20:2, w/w). However, the scavenging activity of ACCH added with EGb was much greater than that of ACCH alone at the same concentration, even greater than that of EGb alone. The half‐effective concentration (IC_50_) of free ACCH and ACCH combined with EGb was 8.87 ± 0.30 and 0.61 ± 0.02 mg/ml, respectively. As shown in Figure 1B, ACCH and the mixture also exhibited excellent scavenging activities against ABTS free radical with IC_50_ of 4.53 ± 0.15 and 0.43 ± 0.02 mg/ml, respectively. Thus, the results indicated that the scavenging free radical activity of ACCH combined with EGb was superior to ACCH alone. EGb may be responsible for improving the antioxidant activity of ACCH. In addition, the results may be also attributed to the interaction between EGb and hydrophobic amino acids in ACCH.

To explore the interactions (synergy, additive or antagonistic/negative) of ACCH and EGb, we conducted isobolographic analysis. The results are shown in Table 3. The mixtures of ACCH and EGb were prepared at a few selected doses (20:1, 20:2 and 20:4, w/w). The observed antioxidant capacity of each mixture was compared with the expected value. It was apparent that ACCH and EGb showed synergism in the three antioxidant assays, and at the ratio of 20:4, the mixture exhibited the strongest synergism in DPPH assays (λ = 0.739), ABTS assay (λ = 0.741) and HO^·^ assays (λ = 0.788). The synergy effect is stronger with the increased amount of EGb in the mixtures, suggesting that EGb played a great role in the synergy of ACCH and EGb mixture.

**Table 3:** Synergistic antioxidant effect for combinations of colla corii asini hydrolysate and ginkgo biloba extracts

Ratio (w/w)	DPPH^·^ (mg/ml)			ABTS^·+^ (mg/ml)			HO^·^ (mg/ml)		
	O	E	λ	O	E	λ	O	E	λ
20:1	0.61 ± 0.02	0.75 ± 0.02	0.813	1.36 ± 0.14	1.54 ± 0.12	0.883	2.75 ± 0.14	3.20 ± 0.22	0.859
20:2	0.32 ± 0.01	0.41 ± 0.01	0.780	0.84 ± 0.08	0.96 ± 0.10	0.875	2.19 ± 0.11	2.60 ± 0.12	0.842
20:4	0.17 ± 0.01	0.23 ± 0.01	0.739	0.43 ± 0.02	0.58 ± 0.02	0.741	1.52 ± 0.07	1.93 ± 0.05	0.788

Data are expressed as means ± standard errors (*n* = 3). O, observed value; E, expected value; λ, interaction index.

### 3.4 Antiproliferation of MCF‐7, MDA‐MB‐231, and L929

Some studies suggested that the damage to cells caused by free radicals, especially the damage to DNA, may play a role in the development of cancer and other health conditions. Natural antioxidants may help lower the risk of developing or dying from cancer in humans by scavenging free radicals in cell (Diplock et al., 1998; Valko et al., 2007). In addition, protein hydrolysates and plant extracts can directly kill cancer cells or induce cell apoptosis (Chi et al., 2015; Pan et al., 2016). The anticancer activity of ACCH, EGb, and their mixture (20:4) was evaluated in this study.

**Figure 2:**
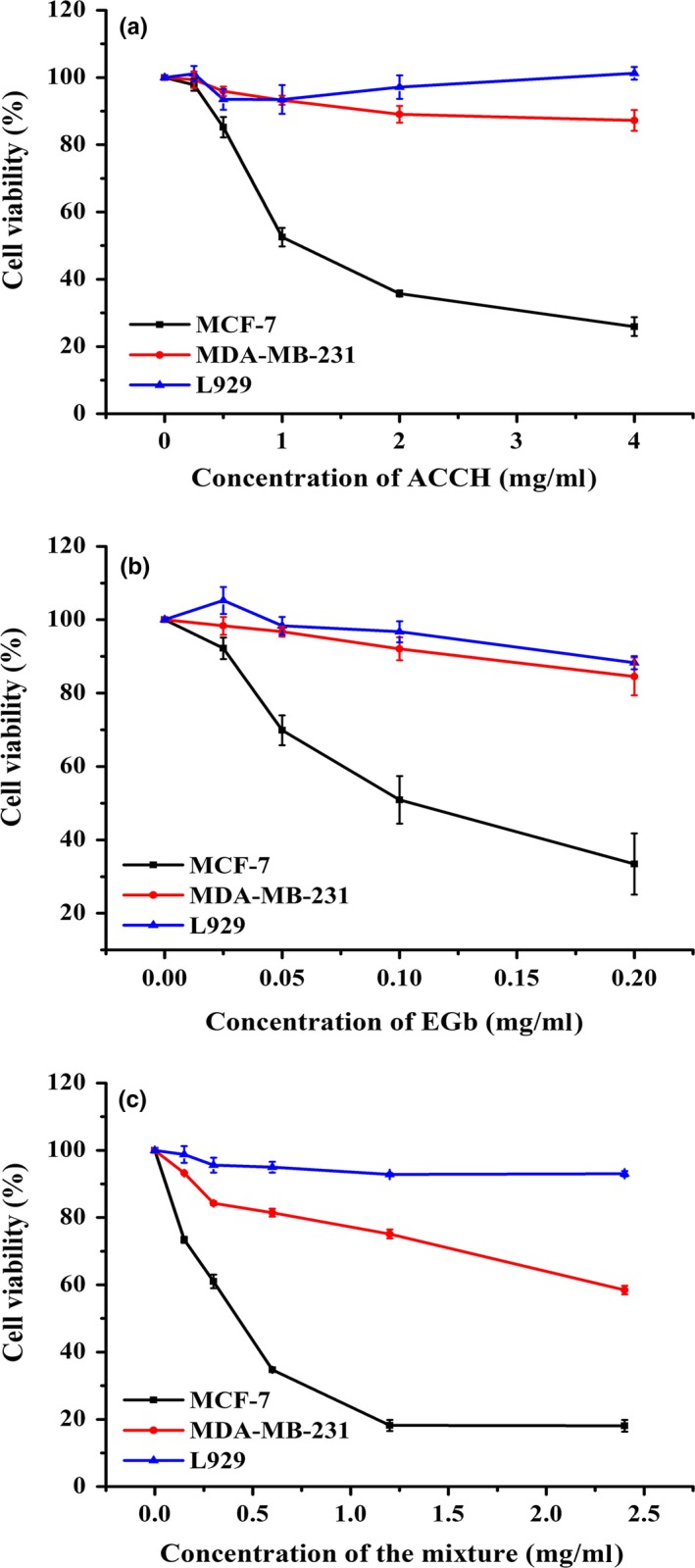
Proliferation inhibition of MCF‐7, MDA‐MB‐231, and L929 cell lines treated with colla corii asini hydrolysates (ACCH) (A), ginkgo biloba extracts (EGb) (B), ACCH: EGb = 20:4 (C). All data are presented as the mean ± *SD* of triplicate results

Cell viability of MCF‐7, MDA‐MB‐231, and L929 treated with ACCH, EGb, and their mixture for 72 hr is shown in Figure 2A, B and C, respectively. ACCH had hardly any cytotoxicity on normal mouse fibroblast cell line L929 and even slightly promoted proliferation of L929 cells (Figure 2A). EGb had hardly any cytotoxicity on L929 in the concentration of 0–0.1 mg/ml (Figure 2B), but showed low cytotoxicity on L929 with cell viability of 88.3 ± 1.78% in the concentration of 0.2 mg/ml. By contrast, the cell viability for L929 with the mixtures of ACCH and EGb was above those with EGb for 72 hr. This indicates that the mixture was cell selective and destroyed only tumor cells and not normal cells (Figure 2C).

Moreover, we observed that cells viability of MCF‐7 and MDA‐MB‐231 decreased in a dose‐dependent manner when the cells were treated with ACCH, EGb and the mixture, respectively, suggesting these three samples all had proliferation inhibitory effect on MCF‐7 and MDA‐MB‐231 cells. For comparison purposes, the IC_50_ values of MCF‐7 and MDA‐MB‐231 cell lines were calculated. After EGb was added, ACCH possesses greater cytotoxicity for MCF‐7 cell lines than ACCH alone, with IC_50_ of 0.39 ± 0.01 and 1.60 ± 0.03 mg/ml, respectively. Meanwhile, the proliferation inhibition of MDA‐MB‐231 was also greatly improved when ACCH was mixed with EGb and the IC_50_ value increased from 23.10 ± 1.63 mg/ml to 4.32 ± 0.53 mg/ml.

The cell selectivity and susceptibility to lysis might be associated with the compositions of cell membrane bilayers and the distribution of phospholipids (Wang et al., 2008). The content of phosphatidylserine (PS) in the outer leaflets of cancer cells is higher than that in the inner leaflets of normal cells in the membranes (Leuschner & Hansel, 2004). Hydrophobic amino acids could increase interaction between antioxidants and the outer leaflets of tumor cell membrane bilayers that have high phospholipid contents (Chi et al., 2015). This may explain the reason that ACCH has cell selectivity. Various flavonoids contained in EGb could be toxic allergens, which lead to certain degree of cytotoxicity of EGb on L929 at 0.2 mg/ml. In addition, ACCH, EGb, and their mixture showed anticancer activity, in accordance with antioxidant activity.

### 3.5 FT‐IR analysis

**Figure 3:**
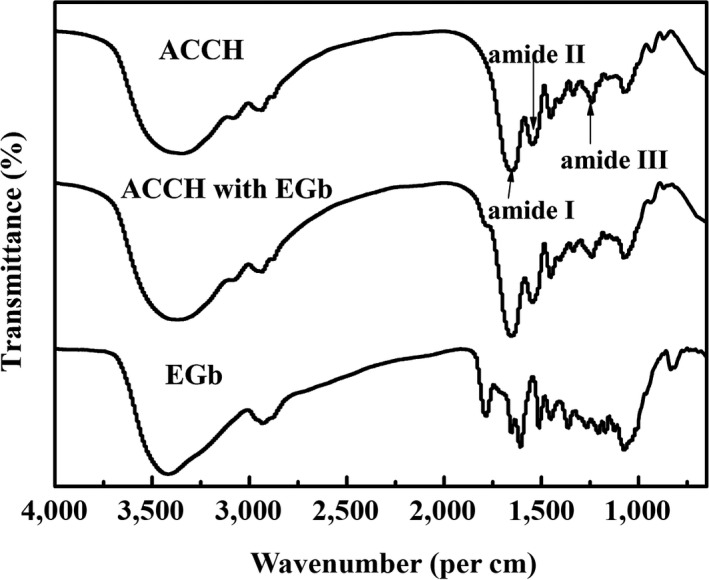
The FT‐IR spectra of colla corii asini hydrolysates (ACCH), ginkgo biloba extracts (EGb), and ACCH with EGb

The FT‐IR spectra of free ACCH, EGb, and ACCH addition of EGb are shown in Figure 3. The broad absorption band of EGb appearing at 3,419, 2,933, and 1,654 cm^−1^ can be attributed to –OH stretching vibration, asymmetric –CH stretches of methylene groups and C=O stretching vibration, respectively. The bands at 1,607 and 1,512 cm^−1^ are corresponding to benzene ring backbone stretching vibrations. For ACCH, the absorption bands at approximately 1,655, 1,546, and 1,240 cm^−1^ were attributed to the C=O stretching (amide I), N–H bending (amide II), and C‐N stretching (amide III), respectively. Compared with that of ACCH, the peak relating to the stretching vibration of N–H bands (3,250 cm^−1^ and 3,600 cm^−1^) of ACCH with EGb became wider and sharper. Moreover, ACCH combined with EGb showed increased intensity of C=O stretching (1,654 cm^−1^) and N–H bending (1,547 cm^−1^). This is mainly because the C=O bands and N–H bands can easily form the intermolecular hydrogen bond with the hydroxyl groups of EGb. What is more, EGb had not only O‐H bands, but also C=O bands directly strengthening the stretching vibration of the related bands. By comparison, we found that no peaks disappeared or appeared in Figure 3, indicating that no new material was found and the existing material did not disappear when ACCH combined with EGb.

## 4 CONCLUSION

In summary, ACCH and EGb exhibited significant synergistic interaction regarding their antioxidant activities and a mixture of 20:4 (ACCH:EGb by weight) displayed the greatest synergistic antioxidant effect among the samples tested (interaction index, λ = 0.739, 0.741, and 0.788, respectively) in DPPH, ABTS and HO^·^ scavenging activity assays. Moreover, the combination of ACCH and EGb possesses stronger cytotoxicity on MCF‐7 cell lines than ACCH alone, with IC_50_ of 0.39 ± 0.01 and 1.60 ± 0.03 mg/ml, respectively. In addition, the mixtures were cell selective and destroyed only tumor cells and not normal cells. Our findings suggest combining ACCH and EGb can serve as better natural sources of antioxidant and anticancer agents, which may find potential uses in the nutraceutical and pharmaceutical applications.

## CONFLICT OF INTEREST

The authors declare no conflict of interest.
